# Pre-cryoablation Embolization of Renal Tumors: Decreasing Probes and Saving Loads

**DOI:** 10.7759/cureus.3676

**Published:** 2018-12-03

**Authors:** Taylor S Harmon, Jerry Matteo, Travis E Meyer, Joanna Kee-Sampson

**Affiliations:** 1 Radiology, University of Texas Medical Branch, Galveston, USA; 2 Radiology, University of Florida College of Medicine, Jacksonville, USA

**Keywords:** interventional radiology, cryoablation, renal cell carcinoma, embolization, percutaneous tumor ablation, tumor, computed tomography, tumor burden, tumor shrinkage, cost effective

## Abstract

The use of adjuvant pre-ablation embolization for renal tumors has been reported in endophytic, centrally located lesions to reduce the risk of injuring the renal collecting system during subsequent cryoablation. In this technical report, we present another utilization of adjuvant pre-ablation embolization, applied for the purpose of decreasing the number of cryoablation probes needed in the ablation intervention. This novel procedural protocol not only decreases the cost of the procedure, but also preserves more normal renal parenchyma, and decreases the risk of injuries related to probe positioning.

## Introduction

Surgical and percutaneous interventional management of renal cell carcinomas has been on the rise, in parallel to the incidental discovery of these tumors from an increase in utilization of cross-sectional imaging [1]. Though the current standard of management for renal cell carcinomas is either partial or total nephrectomy, percutaneous ablation of renal cell carcinomas is both a cost-effective and potentially curative option. Specifically, the cure rate of cryoablation for stage 1A and stage 1B renal cell carcinomas is 97% at five years [2]. Accompanying the increase in the number of percutaneous cryoablation cases is the increase in diversity of case presentations, necessitating refinement in techniques for tumor ablation [3]. Matteo et al. described the use of pre-ablation embolization to reduce the risk of injuring the renal collecting system and surrounding parenchyma when performing cryoablation on endophytic, centrally located tumors [4]. This novel, two-step approach was shown to cause tumor shrinkage, allowing safe and effective percutaneous cryoablation of the lesions [4].

Here we present another utilization of the aforementioned two-step approach, in a patient with limited renal reserve who presented for treatment of a partially endophytic renal lesion. The size and location of the lesion would have required treatment with four cryoablation probes, but in the interest of preserving as much normal renal parenchyma as possible as well as minimizing injury to the renal collecting system, the lesion was embolized prior to cryoablation. After embolization, the tumor was successfully treated with two cryoprobes.

## Technical report

**Figure 1:**
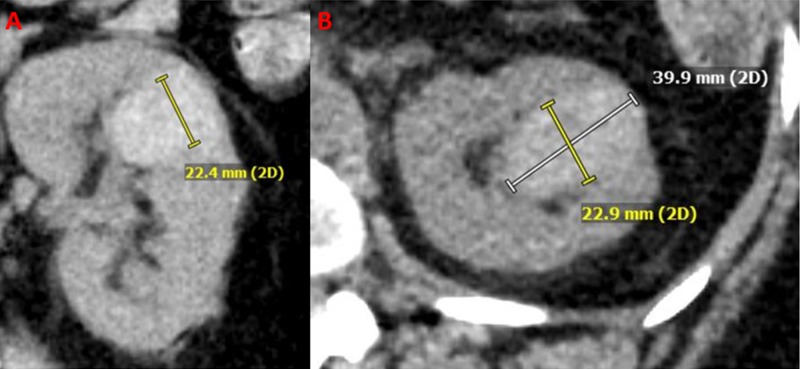
Initial computed tomography (CT). Initial coronal (A) and axial (B) CT images of the renal lesion are shown.

A 51-year-old woman with stage 4 chronic kidney disease (CKD), who was a poor surgical candidate due to multiple co-morbidities, was referred to interventional radiology for the treatment of an endophytic left renal upper pole mass (Figure 1).

Due to the concern for renal collecting system injury as a result of the endophytic tumor morphology and excessive injury to the normal renal parenchyma with the number of probes needed to treat the lesion, a pre-ablation embolization was performed in an attempt to shrink the lesion prior to cryoablation.

Pre-ablation transarterial embolization

**Figure 2:**
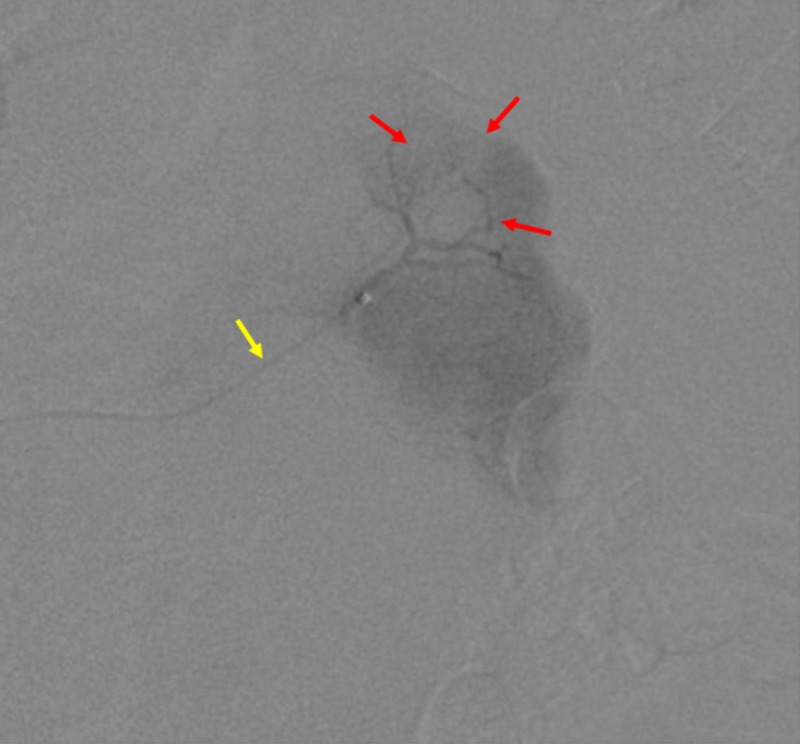
Pre-cryoablation embolization of renal arterial branches. Pre-cryoablation angiography through a microcatheter (yellow arrow) is shown. The red arrows show the tumor outline.

As the patient was in CKD stage 4, an angiography was initially performed with carbon dioxide rather than iodinated contrast to identify the left renal artery and first-order branches. The feeding vessels supplying the left renal mass were then identified on angiography with a small amount of dilute iodinated contrast through a microcatheter (Figure 2).

After super selecting the tumor feeding vessels with the microcatheter, the tumor was embolized with 355-500 micron polyvinyl alcohol particles mixed in dilute iodinated contrast under fluoroscopic guidance. Embolization proceeded until there was no longer any enhancement of the tumor and truncation of the feeding vessels was visualized.

**Figure 3:**
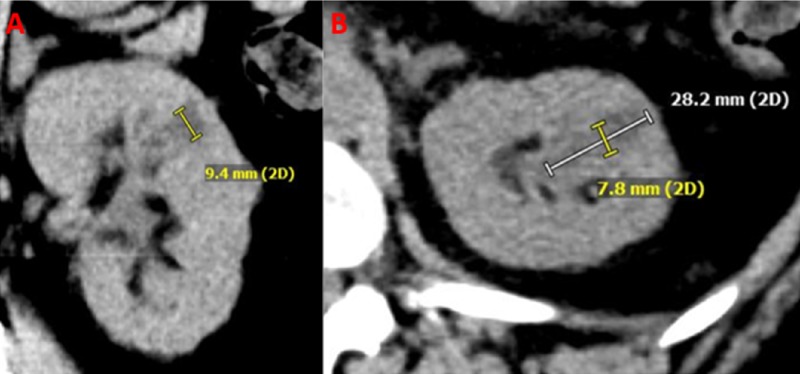
Tumor reduction in the post-embolization period. Coronal (A) and axial (B) images from a follow-up computed tomography (CT) three months post-embolization, shows approximately 90% reduction in the volume of the renal lesion.

A noncontrast computed tomography (CT) was obtained three months after embolization, which showed the renal tumor to have decreased significantly in size (Figure 3).

CT-guided percutaneous cryoablation

**Figure 4:**
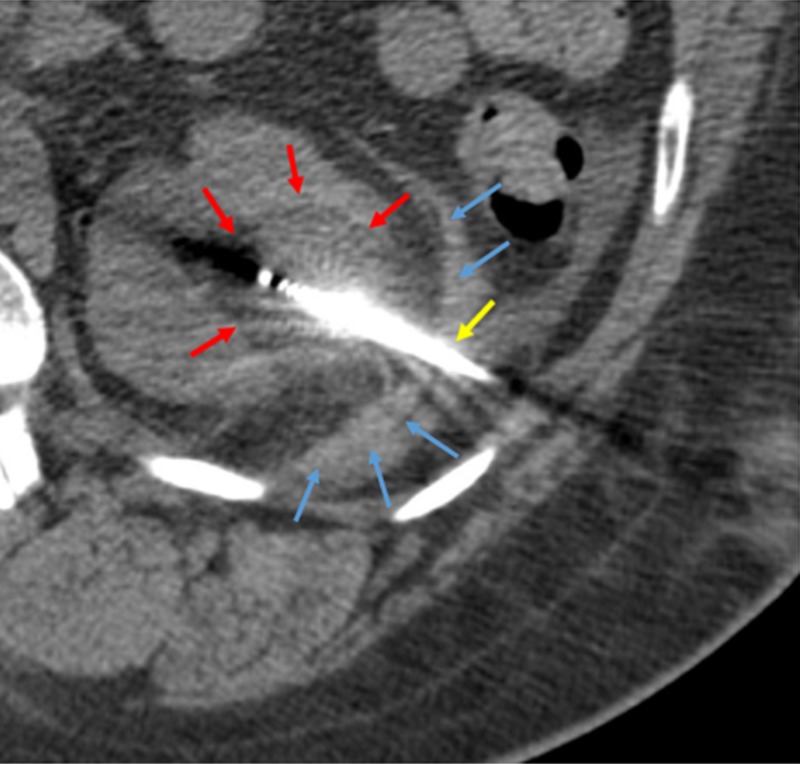
Post-embolization cryoablation of renal cell carcinoma. The hypoattenuated area (red arrows) corresponding to ice ball formation around the tumor is shown. One of the cryoprobes is visualized (yellow arrow). Hydrodissection fluid is seen in the paranephric space (blue arrows).

Three months following embolization, the patient returned for cryablation of the renal lesion. Hydrodissection was utilized in the region of the left retroperitoneum to mobilize the colon away from the paths of the cryoablation needles. Using CT guidance, two 17-gage Ice-Pearl® (Galil Medical, MN, USA) cryoablation probes were inserted into the left renal mass, bracketing the tumor. One probe was placed at the superior margin of the tumor, and the second one was placed at the inferior margin of the tumor, approximately 1 cm apart (Figure 4).

**Figure 5:**
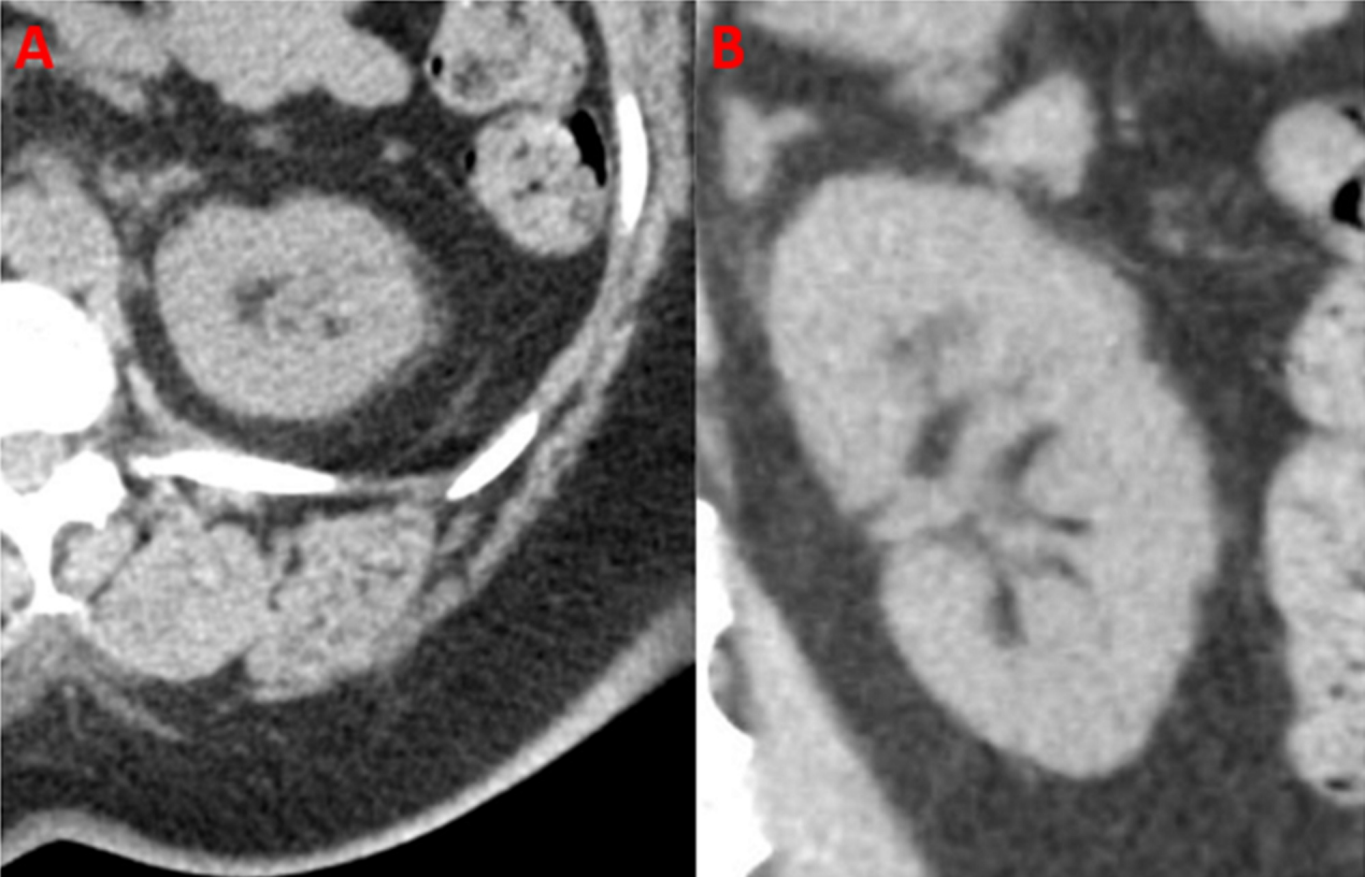
Follow-up computed tomography (CT) after cryoablation. Axial (A) and coronal (B) images show no residual malignancy four months after cryoablation.

Two freeze cycles of 10 minutes each were performed, interspaced by a thaw cycle of five minutes. Intra-procedural CT images demonstrated complete coverage of the lesion by the ice ball. There were no immediate post-procedural complications; the patient tolerated the procedure well. A follow-up CT four months after the cryoablation showed no residual malignancy (Figure 5).

## Discussion

Cryoablation and radiofrequency ablation (RFA) are two of the most commonly utilized modalities for percutaneous ablation of renal tumors. Technical success rates for both cryoablation and RFA are as high as 97%-100%, and have been well reported in the literature [5-8]. Low incidence of residual or recurrent tumors has been reported for renal lesions treated with cryoablation; in a prospective study of 616 patients treated with cryoablation or RFA, the tumors treated with cryoablation had a lower incidence of residual or recurrent tumors (3.9%) compared with RFA (13.4%) [9].

As the rate of percutaneous ablation for renal tumors increases due to increased diagnosis, high technical success rates, promising patient outcomes, and relatively noninvasiveness of the procedures, the issue of cost efficiency deserves recognition. Cryoablation coupled with pre-ablation embolization has already been shown to increase the safety profile of cryoablation for certain tumor morphologies, as demonstrated by Matteo et al. [4]. As presented in the preceding case, it also shows promise in reducing procedural costs.

Currently according to a recently reported cost analysis, the most inexpensive percutaneous tumor ablation method is microwave ablation followed by RFA, with average prices that are comparable (standardized for a 3 cm renal mass) [10]. Cryoablation, though shown to be a highly effective ablative treatment for renal tumors, is more expensive, averaging thousands of dollars more per procedure than RFA or microwave ablation (standardized for a 3 cm renal mass) [10]. Interventional operators can justify the use of cryoablation therapy over RFA and microwave ablation based on high effective cure rates for renal cell carcinomas [2]. However, if pre-ablation embolization of renal cell carcinomas is employed as part of the cryoablation protocol as in the presented case, this may improve the cost efficiency of cryoablation. In the preceding case we presented here, the reduction of tumor volume by 90% as a result of pre-ablation embolization effectively reduced the number of cryoablation probes needed from four to two. Using the aforementioned cost analysis, using two less cryoablation probes decreased the total procedural cost by about 15% [10]. This cost savings show that pre-ablation embolization can be used to improve the cost efficiency of cryoablation by reducing the amount of cryoablation probes needed, as comparable to the cost of RFA and microwave ablation.

A reduction of cryoablation probes used during a procedure can reduce thermal injury to normal renal parenchyma, by decreasing the size of the ice ball. The less probes placed, the lower the risk of complications, such as hemorrhage, pseudoaneurysm formation, pain, and paresthesia at the probe entry site [11].

## Conclusions

Pre-ablation embolization has been previously shown to be an effective complement to cryoablation of large, centrally located renal tumors. This stepwise approach further prevents renal collecting system injury as a result of subsequent cryoablation. The preceding case highlights the additional benefits of cost reduction and renal parenchymal preservation by using pre-ablation embolization in the treatment of renal tumors.

## References

[1] Kay FU, Pedrosa I (2017). Imaging of solid renal masses. Radiol Clin North Am.

[2] Maria T, Georgiades C (2015). Percutaneous cryoablation for renal cell carcinoma. J Kidney Cancer VHL.

[3] (2018). Kidney cancer: introduction. https://www.cancer.net/cancer-types/kidney-cancer/introduction.

[4] Matteo J, Loper T, Hood P, Soule E, Kee-Sampson J, Martin JT (2018). Embolization-induced renal tumor shrinkage followed by definitive cryoablation. Cureus.

[5] Maybody M (2010). An overview of image-guided percutaneous ablation of renal tumors. Semin Intervent Radiol.

[6] Atwell TD, Farrell MA, Leibovich BC, Callstrom MR, Chow GK, Blute ML, Charboneau JW (2008). Percutaneous renal cryoablation: experience treating 115 tumors. J Urol.

[7] Boss A, Clasen S, Kuczyk M (2005). Magnetic resonance-guided percutaneous radiofrequency ablation of renal cell carcinomas: a pilot clinical study. Invest Radiol.

[8] Miki K, Shimomura T, Yamada H, Kishimoto K, Ohishi Y, Harada J, Egawa S (2006). Percutaneous cryoablation of renal cell carcinoma guided by horizontal open magnetic resonance imaging. Int J Urol.

[9] Gervais DA, McGovern FJ, Wood BJ, Goldberg SN, McDougal WS, Mueller PR (2000). Radio-frequency ablation of renal cell carcinoma: early clinical experience. Radiology.

[10] Astani SA, Brown ML, Steusloff K (2014). Comparison of procedure costs of various percutaneous tumor ablation modalities. Radiol Manage.

[11] Allen BC, Remer EM (2010). Percutaneous cryoablation of renal tumors: patient selection, technique, and postprocedural imaging. Radiographics.

